# Supplementary material for: Wearable Technologies for Pediatric Patients with Surgical Infections—More than Counting Steps?
Source: Biosensors (Basel). 2022 Aug 12;12(8):634. doi: 10.3390/bios12080634 (PMC9405945; doi:10.3390/bios12080634)

Table S1. Vital signs recording in study population by wearable device or conventional measurement

ID	Sex	Age	Diagnosis	Total study time	Body temperature								Heart rate								Oxygen saturation							
					Everion® wearable device			Conventional measurements			Difference		Everion® wearable device			Conventional measurements			Difference		Everion® wearable device			Conventional measurements			Difference	
					mean	std	#	mean	std	#	Abs	Rel (%)	mean	std	#	mean	std	#	Abs	Rel (%)	mean	std	#	mean	std	#	Abs	Rel (%)
001	m	14	appendicitis	5d (128h)	35.7	1.1	311236	36.5	0.3	22	-0.8	-2.3	64.7	14.8	311236	63.0	7.5	12	1.7	2.7	94.5	1.9	136502	98.0		1	-3.5	-3.6
002	m	17	appendicitis	2d (64h)	35.5	0.9	215999	37.1	0.4	33	-1.6	-4.4	77.3	12.7	215999	74.4	11.7	10	2.9	4.0	96.9	1.3	85686	98.0	0.7	10	-1.1	-1.1
003	m	13	appendicitis	1d (43h)	34.9	1.4	148722	36.8	0.3	17	-1.9	-5.1	74.3	16.6	148722	77.4	6.1	7	-3.2	-4.1	97.2	1.2	1336	97.4	0.5	7	-0.2	-0.2
004	f	7	osteomyelitis	2d (70h)	35.7	0.9	228905	37.3	0.6	30	-1.6	-4.3	94.2	15.8	228905	108.0	5.2	4	-13.8	-12.8	99.3	0.8	41288	98.5	0.7	2	0.8	0.9
005	m	8	osteomyelitis	2d (69h)	35.5	1.2	229670	36.9	0.6	24	-1.4	-3.8	91.5	15.1	229670	100.0	6.2	3	-8.5	-8.5	99.3	0.8	8330	98.0	0.0	2	1.3	1.3
006	m	10	appendicitis	5d (123h)	35.9	1.2	367225	37.3	0.3	26	-1.4	-3.8	72.4	11.7	367225	69.6	7.7	21	2.8	4.0	98.1	1.7	5135	98.0	0.6	16	0.1	0.1
007	m	8	septic arthritis	1d (43h)	35.0	1.6	83089	37.6	0.7	18	-2.6	-7.0	75.0	13.7	83089			0					0			0		
008	m	8	appendicitis	1d (45h)	33.7	2.0	29394	36.9	0.3	17	-3.2	-8.7	93.5	12.0	29394	106.5	9.1	4	-13.0	-12.2			0	95.8	0.5	4		
009	m	10	osteomyelitis	4d (119h)	35.0	1.2	251609	37.1	0.7	21	-2.1	-5.6	78.9	15.0	251609			0			98.0	1.4	5321			0		
010	m	9	appendicitis	0d (21h)	35.7	0.8	73791	37.1	0.4	8	-1.4	-3.7	83.5	10.2	73791			0			98.3	1.3	3932			0		
011	m	7	septic arthritis	0d (10h)	35.2	1.1	22042	36.8	0.4	6	-1.5	-4.2	111.7	13.6	22042			0			97.1	1.1	417			0		
012	f	15	appendicitis	1d (45h)	35.2	1.5	158047	36.7	0.2	10	-1.5	-4.2	68.5	11.1	158047	74.0		1	-5.5	-7.5	95.6	1.4	25455	96.0		1	-0.4	-0.4
013	f	4	appendicitis	2d (67h)	34.5	1.0	201450	37.1	0.7	14	-2.6	-6.9	94.3	18.4	201450	101.3	15.0	6	-7.1	-7.0	98.1	1.0	16442	98.0	0.0	3	0.1	0.1
014	m	4	septic arthritis	1d (33h)	35.6	1.0	70250	37.1	0.5	20	-1.5	-4.0	104.5	19.9	70250	115.0	24.6	11	-10.5	-9.2	98.1	1.2	5466	96.7	1.8	10	1.4	1.4
015	f	11	appendicitis	0d (18h)	36.4	0.8	63936	36.7	0.2	7	-0.2	-0.6	69.5	9.1	63936	66.0		1	3.5	5.3			0	96.0		1		
016	f	14	appendicitis	0d (19h)	35.0	1.4	67607	37.1	0.8	15	-2.0	-5.5	72.3	11.2	67607	92.3	13.0	4	-20.0	-21.6	98.2	1.4	22181	100.0		1	-1.8	-1.8
017	m	7	appendicitis	0d (17h)	35.3	1.1	60474	37.0	0.4	7	-1.7	-4.6	93.0	16.1	60474			0					0			0		
019	f	4	osteomyelitis	0d (23h)	35.4	0.6	29281	36.8	0.4	6	-1.4	-3.8	111.5	16.3	29281	80.0		1	31.5	39.4			0			0		

020	m	6	appendicitis	2d (55h)	35.5	1.8	107073	37.7	0.7	38	-2.2	-5.8	113.8	17.7	107073	110.4	14.4	14	3.4	3.1	97.2	1.7	3288	98.7	0.7	9	-1.4	-1.4
021	m	15	appendicitis	0d (17h)	35.4	0.9	61246	36.6	0.2	5	-1.3	-3.5	52.2	9.3	61246			0			97.1	1.4	44741			0		
										Mean	-1.7	-4.6						Mean	-2.5	-1.7						Mean	-0.4	-0.4
										STD	0.6	1.7						STD	12.3	14.3						STD	1.5	1.5

[1] Abbreviations: Abs, absolute; rel, relative; d, days; h, hours; f, female; m, male; STD (std), standard deviation

Figure S1. Everion® device, worn by a 3-year-old child.

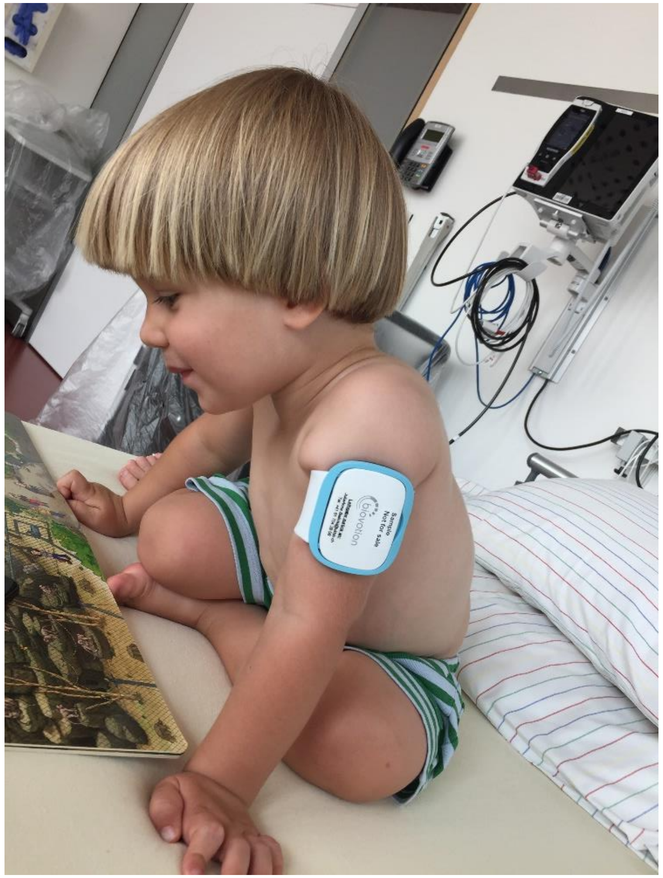

Supplement: Supplementary file 1 [file biosensors-12-00634-s001.zip › biosensors-1821909-supplementary.pdf]
